# Live triatomine bug, vector of *Trypanosoma cruzi* , found engorged in Lisbon hotel room: A first for Portugal and for Europe

**DOI:** 10.1186/s13071-026-07464-4

**Published:** 2026-06-14

**Authors:** Jennifer K. Peterson, Alexander R. Kelley, Trinity Antoszewski, Madeline Brown, Hanna Cortes, Peyton I. Easton, Grace Ferry, Thoburn Freeman, Cate Freiwald, Erik Hagen, Hunt Kinnaird, Luna Lewin, Malaki Lewis, Jessie McNulty, Natalie Moore, Erin Mullis, Stella Pettit, Luke Schultz, Sarah Sharp, William Stocker, Jillian Tunstall, Jader de Oliveira

**Affiliations:** 1 Department of Entomology and Wildlife Ecology, University of Delaware, Newark, DE USA; 2 School of Pharmaceutical Sciences, São Paulo State University (UNESP), Araraquara, São Paulo Brazil; 3 Department of Entomology, National Museum of Natural History, Smithsonian Institution, Washington, DC USA

**Keywords:** Accidental vector importation, Vector hitchhiking, *Hospesneotomae protracta*, *Triatoma protracta*, Triatomine bugs, *Trypanosoma cruzi*, Europe, Portugal, Chagas disease

## Abstract

Triatomine bugs are blood feeding insects that transmit the parasite *Trypanosoma cruzi*, causative agent of Chagas disease. The bugs are found primarily in the Americas with a few species in Asia and Africa. Here, we report the first case of a live triatomine bug in Europe, found in a Lisbon hotel room. In August 2025 the hotel room occupants discovered a triatomine bug perched on the headboard of their bed. Upon capture, bright red blood emerged from the bug; the occupants suspected that it had bitten them during the night. The bug was identified morphologically as triatomine species *Hospesneotomae protracta*, which was confirmed molecularly. *Hospesneotomae protracta* is native to the southwestern USA and northern Mexico, where it is a competent *T. cruzi* vector. *Trypanosoma cruzi* was not detected in this specimen. Although this case likely represents an accidental importation, it illustrates the ease with which disease vectors can be unknowingly transported globally. Ergo it is crucial to document and share these findings to prevent introductions of non-native arthropods of medical importance.

**Graphical Abstract:**

**Figure:**
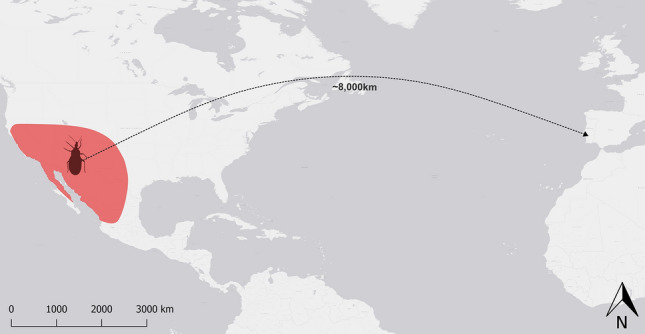

**Supplementary Information:**

The online version contains supplementary material available at 10.1186/s13071-026-07464-4.

## Background

Triatomine bugs (Hemiptera: Reduviidae: Triatominae) are obligate hematophagous insects found primarily in the New World [1]. Commonly known as “kissing bugs,” the insects are of public health significance because they vector *Trypanosoma cruzi,* the causative agent of Chagas disease. Chagas disease is a chronic illness that, when left untreated, can lead to serious cardiac and/or gastrointestinal alterations [2]. An estimated 6–7 million people worldwide are infected with *T. cruzi* [3].

Triatomine bug diversity and abundance are concentrated in tropical and subtropical regions of the Americas (referred to as “New World” species [3]). A handful of triatomine bug species are found in in Africa, Asia, Australia, and the South Pacific (“Old World” species), but not in continental Europe [4]. Notably, Old World Triatominae are not believed to harbor or transmit *T. cruzi*. The sole report of a triatomine bug in Europe occurred in 2022 when a single dead Old World specimen of *Triatoma rubrofasciata* was found in Spain in a commercial shipment from China [5]. Here, we present the first case of a live, free-living New World triatomine bug species in Europe, which was found engorged with fresh blood in a luxury hotel room in Lisbon, Portugal. The specimen was likely imported accidentally and does not represent an introduced European triatomine bug population. Nonetheless, the case is illustrative of the ease with which disease vectors can be unknowingly transported globally.

### Case description

**Fig. 1:**
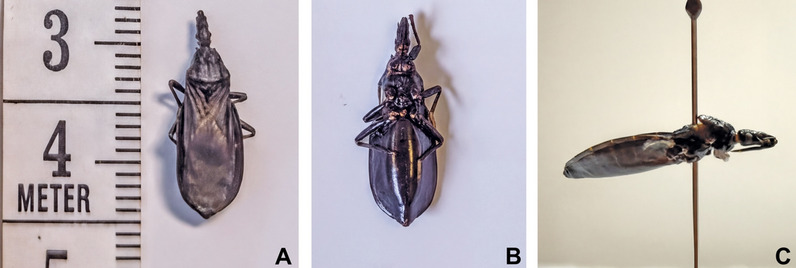
Dorsal (**A**), ventral (**B**), and lateral (**C**) views of the *Hospesneotomae protracta* specimen found in Lisbon hotel room

On the morning of 26 August 2025, a live triatomine bug (Fig. 1, Additional file 1: Fig. S1) was found in a tenth floor luxury hotel room in Lisbon, Portugal. The bug was found alive on the headboard of the hotel bed, and upon capturing the insect with a tissue, red blood emerged from below its pronotum, suggesting that it had recently fed (Additional file 1: Fig. S1). The bug was discovered by the hotel room occupants, a couple visiting Lisbon from the east coast of the USA. They were in Lisbon as part of a pre-trip extension prior to taking a river cruise. They had arrived the day before (25 August) and spent one night in the room. They believed that one of them had been bitten by the bug during the night because one of them had woken up with a dark brown streak on their arm and finger, which they believed to be the insect’s feces.

The couple reported the incident to the hotel desk receptionist and hotel manager, showing them photographs of the bug (Additional file 1: Fig. S1). The hotel staff apologized and moved the couple to a different room. They stayed in the hotel (in a different room) for three additional nights and did not encounter any more triatomine bugs.

The hotel was in high-rise building in an upscale area of Lisbon. The room occupants who found the bug reported that they observed several other cruise guests from the USA staying in the same hotel, suggesting that it is an establishment that caters to international tourists. They reported the condition of the hotel as “nice but needing cleaning.” The hotel is advertised on its website as a five-star establishment.

Upon returning to the USA, the couple reached out to their primary care physician because they were concerned about possible *T. cruzi* exposure and Chagas disease. The physician dismissed their concerns, after which they requested assistance from the Infectious Disease Entomology across Scales (IDEAS) lab in the Department of Entomology and Wildlife Ecology at the University of Delaware. The couple sent the specimen to the IDEAS lab, where it was identified to species and tested for *T. cruzi* infection. They also consulted an infectious disease specialist about 6 weeks after the incident, who ordered a script for the couple to be screened for *T. cruzi* antibodies. The test was performed at a commercial diagnostic laboratory (Labcorp). Available information for Labcorp indicates that they use the Alinity s Chagas testAlinity s Chagas [6], which is a chemiluminescent microparticle immunoassay (CMIA) manufactured by Abbott™ to detect antibodies against *T. cruzi* in human serum and plasma. Antibodies were not detected in either spouse.

Despite having undergone two transatlantic journeys, the specimen was relatively intact, with just its antennae and parts of the legs missing or loose (Fig. 1; all legs fell off during positioning for lateral view photo). The specimen was identified as an adult female *Hospesneotomae protracta* Uhler, 1894 (formerly *Triatoma protracta* [7]). The species is native to the southwestern USA and Northwest Mexico, and an important North American vector of *T. cruzi* [7–13]. Species identification was made using a consistent set of external morphological characters, following the classical keys by Lent and Wygodzinsky [14], the updated taxonomic framework proposed by Paiva et al. [7], and relevant data from Valenzuela et al. [10]. In brief, the identification was based on body size (males 13–19.5 mm, females 15–23 mm), a trapezoidal pronotum that is smooth and laterally carinate with a slightly elevated anterior lobe lacking tubercles, and a rugose posterior lobe with rounded humeral angles. The head is about twice as long as wide and distinctly convex dorsally and shows a clear arcuate transverse impression at the base of the clypeus, visible in both dorsal and lateral views. The anteocular region is much longer than the postocular region, the genae taper apically without reaching the clypeus, and the eyes are relatively small, reaching the level of the upper or lower surface of the head in lateral view. Antenniferous tubercles are positioned near the middle of the anteocular region.

Molecular confirmation of the species was performed using polymerase chain reaction (PCR) and Sanger sequencing, as described previously [15–17]. DNA extracted from the insect’s lower abdomen was amplified in a conventional PCR using the cytb-intF/R primers (5′-YCT ACT ATC CGC GGT TCC TTA-3′ and 5′-ATA CTA TTG CAA TTA CTC CTC CTA-3′, respectively), which amplify a 443 base pair fragment of the triatomine mitochondrial cytochrome *b* (*cytb*) gene. Amplicons were Sanger sequenced and sequence files were processed in Geneious Prime 2022.0.1 (https://www.geneious.com). Primers and low-quality bases were trimmed from the forward and reverse reads using the “Trim Ends” function with default settings. Sequences were aligned using the “De Novo Assemble” function with default settings and the forward and reverse sequences were merged using the “Generate Consensus Sequence” option. The resulting consensus sequence was 398 bp. The sequence was aligned to similar sequences in Genbank using the BLAST function [18]. The top ten alignments matched the species *H. protracta* with the top hit (MT556662.1) having 97.99% identity, 100% query cover, and an *e*-value of 0.0.

**Fig. 2:**
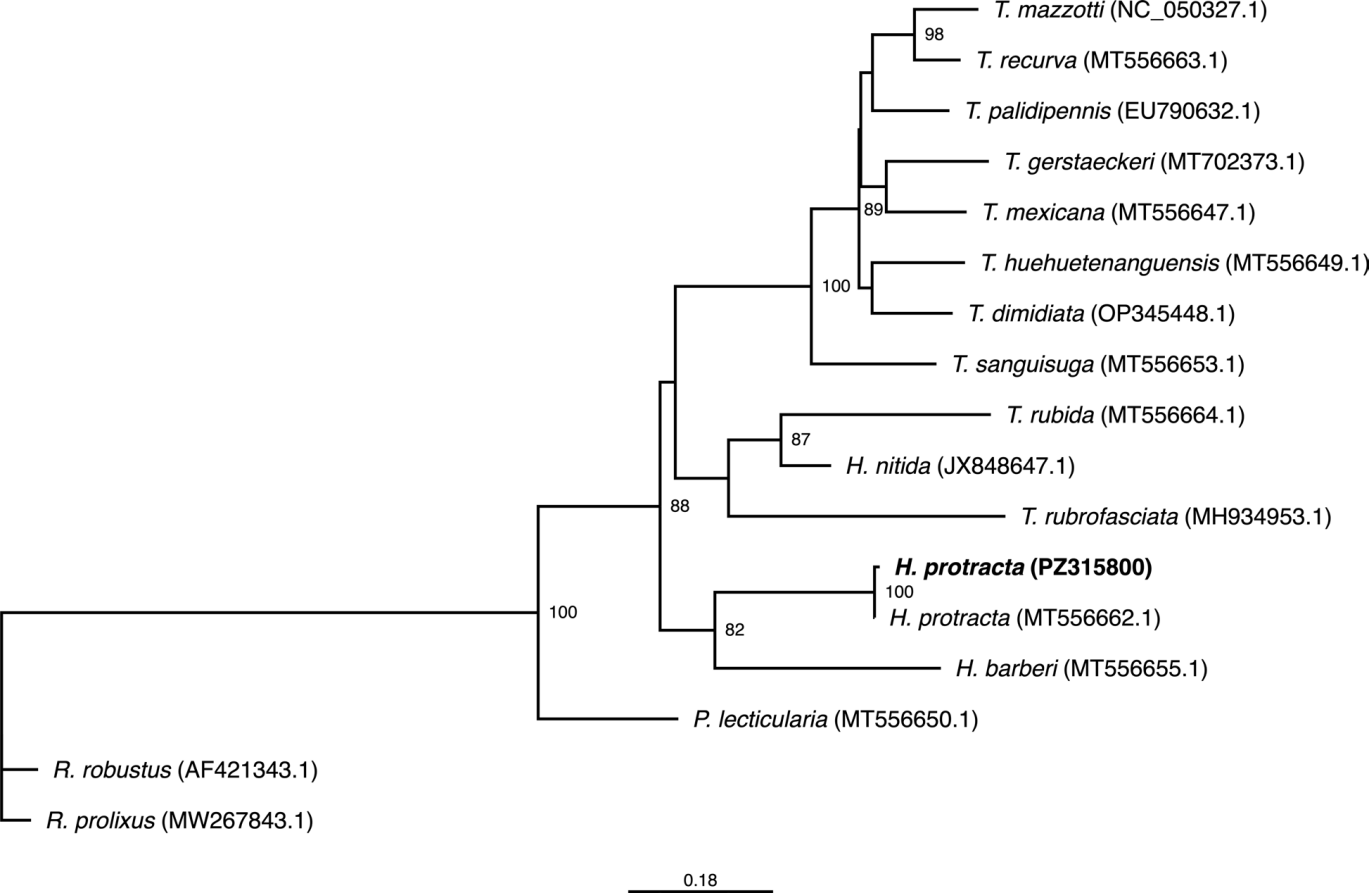
Maximum likelihood phylogeny of Triatomine species based on *cytb* gene sequences. The specimen sequenced in this study (in bold) clusters with *H. protracta*. Node labels represent bootstrap support percentages. The scale bar represents the number of nucleotide substitutions per site. Genbank accession numbers are in parentheses. Abbreviated genera shown in the tree are as follows: *Hospesneotomae* (H); *Paratriatoma* (P); *Rhodnius* (R); and *Triatoma* (T)

In a phylogenetic tree generated with the sequence, the specimen clustered with *H. protracta* with 100% confidence (Fig. 2). To create the phylogeny, reference sequences were downloaded from the National Center for Biotechnology Information (NCBI)’s Nucleotide database using EDirect v19.0 utilities “esearch” and “efetch” [19] and imported into Geneious. Complete mitochondrial sequences were manually trimmed to the *cytb* gene boundaries as defined by the NCBI reference annotations. The consensus sequence was aligned with trimmed references using the Clustal Omega Geneious plug-in v1.2.3 using the default automatic setting (corresponding to --auto on the command line [20]). A phylogeny was inferred using IQ-TREE v3.1.0 [21] with outgroup taxa specified (*Rhodnius robustus* and* R. prolixus*). Bootstrap values were computed using ultrafast bootstrap approximation (UFBoot) with 10,000 replicates [22] and ModelFinder was used to determine the best-fit model [23]. The tree was visualized and customized using Iroki [24].

*Trypanosoma cruzi* DNA was not detected in the specimen, which was screened for the parasite in a duplex quantative PCR (qPCR) run in triplicate [25]. Briefly, DNA extracted from the specimen was amplified using the cruzi1 and cruzi2 primers (5′-AST CGG CTG ATC GTT TTC GA-3′ and 5′-AAT TCC TCC AAG CAG CGG ATA-3′, respectively) and the cruzi 3 probe (5′-FAM-TTG GTG TCC AGT GTG TG-NFQ-MGB-3′), which target a 165 bp sequence of *T. cruzi* satellite DNA. A 163 bp sequence of triatomine 12S ribosomal RNA was targeted in the same reaction using the P2B (5′-AAA GAA TTT GGC GGT AAT TTA GTC T-3′) and P6R (5′-GCT GCA CCT TGA CCT GAC ATT-3′) primers and the Triat probe (5′-VIC-TCA GAG GAA TCT GCC CTG TA-NFQ-MGB-3′) as an internal amplification control (IAC). The IAC successfully amplified triatomine DNA, with a strong positive signal and critical threshold values of 17 for all three replicates, providing further taxonomic confirmation that the specimen was a triatomine bug.

All details and data for the specimen were shared with the Center for Vectors and Infectious Diseases Research (CEVDI) at the Portuguese National Institute of Health Doctor Ricardo Jorge (INSA).

## Discussion

Here, we present the first case of a live, New World triatomine bug species in Europe. We detected and identified *H. protracta*, a species from North America, in a hotel in Lisbon, Portugal. The specimen was likely an accidental importation that was transported overseas passively, which is common for smaller disease vectors such as mosquitoes [26], but just a handful of modern-day reports exist of accidental overseas triatomine bug transport. In pre-Columbian populations, passive dispersal of highly domesticated triatomine species such as *Triatoma infestans* and *R. prolixus* was facilitated by human activities and overland movement [27–30], while *T. rubrofasciata* is hypothesized to have dispersed with ship rats on ancient maritime trade routes [31, 32]. However, modern reports of passive overseas dispersal are rare. In the USA, two *T. ryckmani* individuals were intercepted at the Miami airport in 1970 on a Bromeliad sent from Guatemala [33]. More recently, as mentioned above, a dead *T. rubrofasciata* specimen was discovered in a box of commercial goods in Spain in 2022 [5]. The specimen had hitchhiked to Spain from China, where the triatomine bugs are not believed to harbor *T. cruzi.* Another North American reduviid species, *Zelus renardii*, was first recorded in Europe (in Greece and Spain) in 2010, where authorities suspected that it was introduced via eggs in imported plants [34, 35]. Interestingly, *H. protracta* is one of just a handful of triatomine species that glues their eggs onto the substrate upon which they oviposit, which lends itself to passive dispersal [36]. *Zelus renardii* preys on beneficial and harmful crop insects and is not a human disease vector. The species continues to expand its range in Europe, and it was reported for the first time in Portugal in 2020. Researchers estimate that, since its introduction in 2010, *Z. renardii* has spread in Europe at a rate of 40 km per year [34, 37, 38], illustrating the speed with which introduced insects can spread once the population is seeded.

**Fig. 3:**
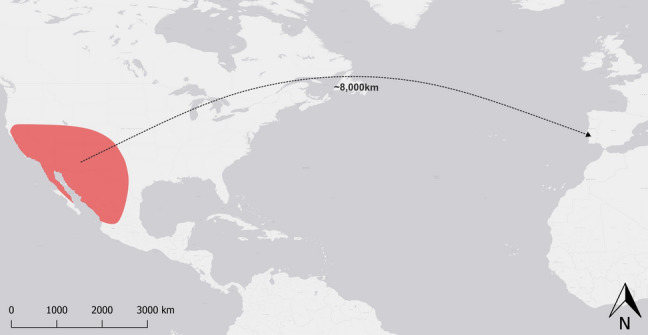
Possible route traveled by *H. protracta* specimen. Red color represents approximate *H. protracta* range, based on observations reported on iNaturalist [55]. Map generated using QGIS (https://qgis.org/).

We cannot ascertain the exact geographical origin of the *H. protracta* specimen or the way in which it was transported to Lisbon, but it probably came from somewhere in the southwestern USA (Fig. 3) and was transported in someone’s personal belongings or a shipment of commercial goods. Portugal is an increasingly popular destination for American tourists and American exports; in 2024, 2.3 million US tourists visited Portugal, up 12% from the previous year, while 244 million dollars of agricultural goods were exported to Portugal from the USA in 2022 [39, 40]. The states of California and Texas, both endemic for *H. protracta,* are among the top six US states with the highest exports to Portugal.

Behavioral characteristics specific to *H. protracta* may have contributed to the realization of the specimen’s transatlantic journey. First, the species is native to arid and semi-arid regions of the southwestern USA and northwestern Mexico, adapted to desert climates with hot summers and cool, dry winters [8, 10]. A 20-year study of *H. protracta* populations in Griffith Park, Los Angeles, CA, found that peak movement occurred during the months of July and August, aligning with the time of year (August) in which the bug was found in Lisbon. These dispersals occurred when temperatures were between 16 and 24 °C (60–76°F; [11]), which is close to the average temperature of the cargo hold of a commercial jet, which is pressurized and kept around 18 °C (65°F; [41]). If the specimen was transported to Portugal in someone’s personal belongings, this adaptation to cool climates may have helped it to survive the 9 h (or more) flight to Lisbon from the southwestern USA or northwestern Mexico. Additionally, the species is closely associated with the grassy, inner breeding cavities of woodrat middens, thus accustomed to tight spaces [42]. Indeed, many triatomine species, including *H. protracta* exhibit a “sit and wait” nest predation strategy where they remain in tiny spaces for long periods of time awaiting their next blood meal [42]. These species have a high starvation capacity (weeks to months), which would also contribute to their survival on long-haul trips and the time spent afterward awaiting a blood meal [43, 44]. When given the opportunity to feed however, *H. protracta* are known to be aggressive feeders, taking advantage of available blood meals in laboratory settings within 30–60 s [45]. In the wild, they will enter human homes and feed opportunistically on humans and their animals, as the specimen described here did, and several reports of *H. protracta* bites exist in its native geographic range [11, 13, 45, 46].

Although *T.* cruzi, was not detected in the *H. protracta* specimen reported here, the species is a competent *T. cruzi* vector . In a study of 1759 *H. protracta* individuals collected in Los Angeles, CA, between 1941 and 1967, Wood et al. [11] observed trypanosomes in the feces of 593 individuals, revealing a prevalence of 33.1%. A PCR-based analysis likely would have revealed an even higher prevalence, as PCR is often more sensitive than microscopy for *T. cruzi* detection in the vector [47, 48]. A more recent study of *T. cruzi* infection in *H. protracta* in California found a prevalence ranging between 18.7% (26/139) and 36.4% (8/22), indicating that the parasite still actively circulates in the region [13].

Despite being competent for *T. cruzi* infection, the likelihood of *T. cruzi* transmission to a human from a single, isolated triatomine bite is estimated to be very low (0.058% [49]). This low likelihood is because *T. cruzi* is transmitted in the vector’s excrement (not its saliva), which requires that the triatomine bug defecate during or shortly after feeding. For *H. protracta*, the risk of *T. cruzi* transmission is considered low due to its infrequent contact with humans [13]. However, a recent study found that about 36% of lab-reared adult *H. protracta* defecated within 1 min of repletion after blood feeding, suggesting that, if contact with a human is made, the species could transmit *T. cruzi* [45]. A recent study of 52 human Chagas cases diagnosed between 2013 and 2023 in California (where the most common triatomine species is *H. protracta*) found that local *T. cruzi* transmission could not be ruled out in 9 of the 52 cases, leaving open the possibility that autochthonous *T. cruzi* transmission to humans occasionally occurs in California [50].

Finally, there is also a very low probability of an imported uninfected triatomine bug acquiring the infection in the nonendemic region. There are an estimated 125,000 people infected with *T. cruzi* in Western Europe [51]. In Portugal, the *T. cruzi* infection prevalence among migrants from Chagas-endemic regions is unknown; the World Health Organization estimate is between 900 and 89,900 [52] and  the Chagas disease underdiagnosis rate is estimated to be over 99% [53]. In addition, climate modeling suggests that several areas of Portugal and Southern Europe are climatically suitable for some triatomine species, should a population be introduced [54]. These data serve to highlight the importance of surveillance for triatomine importations in nonendemic regions.

## Conclusions

The case presented here highlights the ease with which portable and sturdy triatomine species can be passively transported long distances to nonendemic regions. Considering the incidence reported here in the context of ever-increasing global trade and tourism combined with climate change and expansion of habitat suitability for these vectors, this case illustrates the importance of remaining vigilant to the entry, establishment, and spread of potentially harmful insect species.

## Supplementary Information

Additional file 1: Figure S1. Triatomine specimen moments after capture, resting on the tissue used to absorb the blood that emerged from its abdomen.

## Data Availability

Sequence data from this study are available on Genbank under accession number PZ315800.
