# Supplementary figures and images for: Live triatomine bug, vector of Trypanosoma cruzi , found engorged in Lisbon hotel room: A first for Portugal and for Europe
Source: Parasit Vectors. 2026 Jun 14;19:250. doi: 10.1186/s13071-026-07464-4 (PMC13273934; doi:10.1186/s13071-026-07464-4)

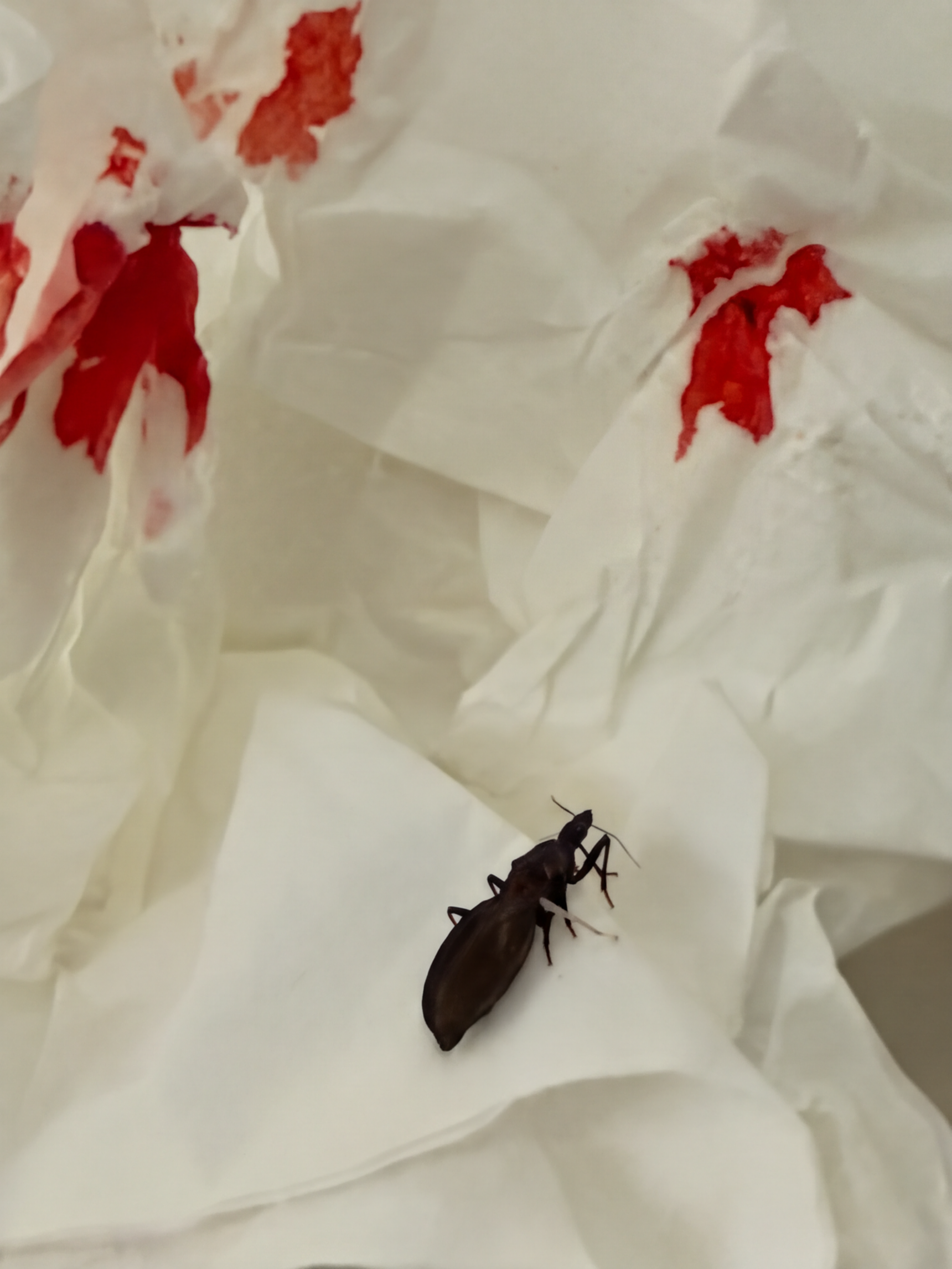

Supplement: Supplementary file 1 — Additional file 1: Figure S1. Triatomine specimen moments after capture, resting on the tissue used to absorb the blood that emerged from its abdomen. [file 13071_2026_7464_MOESM1_ESM.png]
